# Orthopaedic portrayals in *The Seven Works of Mercy* painted by a Dutch master in the year 1504

**DOI:** 10.1007/s00264-025-06653-7

**Published:** 2025-09-26

**Authors:** Stella A. Bult, Pien E.J. de Ruiter, Pieter-Paul A. Vergroesen, Thomas M. van Gulik

**Affiliations:** 1 Dept. of Surgery, Amsterdam University Medical Centers, Amsterdam, Netherlands; 2 Dept. of Orthopaedic Surgery, Noordwest Ziekenhuisgroep, Alkmaar, Netherlands

**Keywords:** Visual arts, Art based learning, Medical humanities, Iconodiagnostics, Orthopaedics

## Abstract

**Purpose:**

We examined *The Seven Works of Mercy*, painted by the Master of Alkmaar in 1504, through the lens of orthopaedic pathology. This study approaches the panels from a medical perspective, aiming to uncover visual indicators of disease and disability. The findings offer insight into how physical abnormalities were observed and depicted in the early sixteenth century. To our knowledge, this is the first study to explore orthopaedic pathology in the *Seven Works of Mercy.*

**Methods:**

An interdisciplinary analysis of *The Seven Works of Mercy *was undertaken, with a focus on the visual representation of illness and physical disabilities. The seven panels were examined to identify physical abnormalities. The findings were compared with clinical features of the suggested illnesses and disabilities and with known medical conditions prevalent in the fifteenth and sixteenth century in Europe.

**Results:**

Several depicted orthopaedic disabilities were suggested in the panels of *The Seven Works of Mercy*. Possible underlying conditions included clubfeet, spinal tuberculosis (Pott’s disease), syphilis, poliomyelitis, ergotism, and genu recurvatum. The physical deformities, depicted with remarkable anatomical detail, were cross-referenced with known clinical presentations. In several cases, assistive devices and posture supported the proposed diagnoses.

**Conclusion:**

*The Seven Works of Mercy* by the Master of Alkmaar is a mirror of society in the early sixteenth century, in which a number of depicted orthopaedic conditions were identified. While artistic interpretation must be considered, several physical deformities and disabilities are reproduced with remarkable detail. The artist captured in this masterpiece, a gallery of orthopaedic pathologies common in his time.

## Introduction

**Fig. 1:**
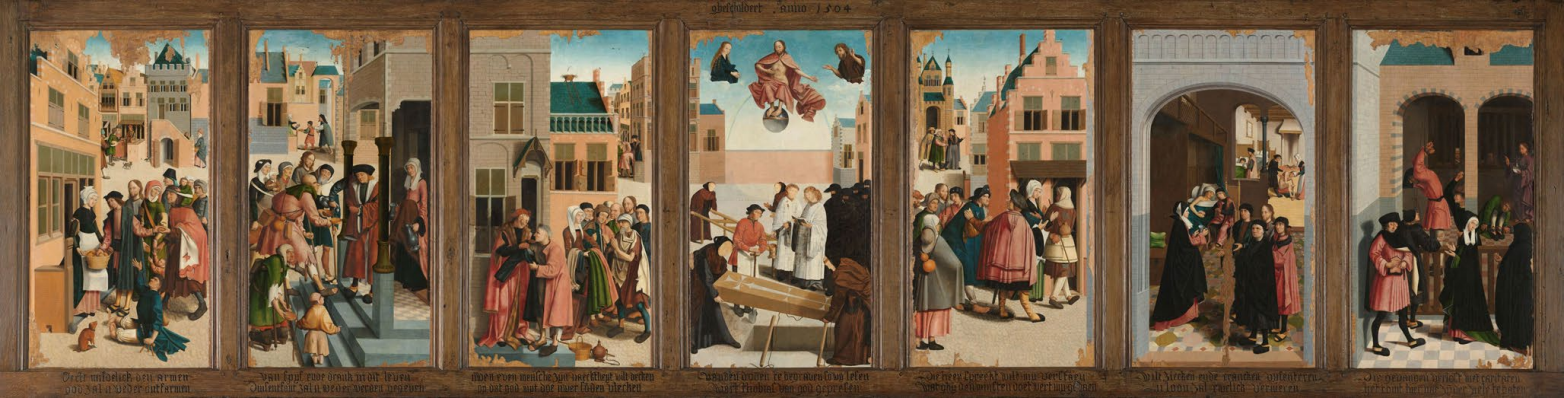
Master of Alkmaar, *The Seven Works of Mercy*, 1504, oil on panel, 119,1 × 469,5 cm, Rijksmuseum, Amsterdam, public domain

In the Christian tradition, The Seven Works of Mercy represent seven ways in which believers are called to show compassion. In this article, we examine *The Seven Works of Mercy*, a Dutch painting by the Master of Alkmaar from 1504 AD through the lens of orthopaedic conditions, see Figure 1. In the seven panels of this artwork, various forms of charity are depicted, including a number of people showing disabilities. This study approaches the panels from a medical perspective, aiming to uncover visual indicators of disease and disability with an emphasis on orthopaedic disabilities. The findings offer insight into the occurrence of physical pathology in the early sixteenth century, in the way it was observed and depicted through the eyes of the artist. To our knowledge, this is the first study to describe orthopaedic pathology in *The Seven Works of Mercy.*

## Materials and methods

*The Seven Works of Mercy* is an artwork consisting of seven paintings on panels, on display in the permanent exhibition of the Rijksmuseum in Amsterdam. The artwork was created in 1504 in Alkmaar, a town in the north-western part of the Netherlands. As the name of the artist is unknown, the painter is referred to as the Master of Alkmaar. The artwork has been extensively restored after severe damage incurred during the Reformation Iconoclasm [1].

An interdisciplinary analysis was conducted of *The Seven Works of Mercy*, with a focus on the visual representation of illness and physical disabilities. By combining an art historical and a medical approach, this study tries to identify possible orthopaedic disabilities depicted in the persons presented across the panels. These observations are situated within the broader context of the early sixteenth century, a period marked by frequent outbreaks of infectious epidemics and chronic poverty. It provides us not only with insight into the medical conditions of that period but also allows us a glimpse into the available medical care and devices.

A key focus of this paper is the presence and interpretation of orthopaedic imagery in this artwork. In this study, historical iconography and contemporary clinical knowledge were combined. The authors’ shared interest and background, combined with their collaboration with the Rijksmuseum, enabled them to conduct direct visual analysis of the artwork. In this study, historical iconography and contemporary clinical knowledge were combined. Through close examination of the panels, possible physical abnormalities were identified among the depicted figures. The diagnoses were systematically evaluated using the 11-step iconodiagnostic method developed by Charlier et al. [2].The findings were compared with contemporary clinical features of the suggested illnesses and disabilities, in conjunction with known medical conditions prevalent in fifteenth and sixteenth century in Europe.

## Results

All panels of the artwork were examined and all possible medical features listed. Following is a detailed account of our findings, see Table 1.

### Feeding the hungry

On the first panel, bread is distributed. The cityscape behind the figures provides a stage on which the rich, members of the Brotherhood of the Holy Spirit, donate food to those in need. The city is clean and tidy and shows an idealized version of the city of Alkmaar in the late Middle Ages.

The person on the foreground in the blue cloth is sitting on the ground. His feet look deformed, and he seemingly isn’t able to walk, using hand crutches to move around. The way his feet are rotated inwards and downwards is characteristic of untreated clubfeet, also called *congenital talipes equinovarus* [3, 4]. Today, if children are born with signs of clubfeet, immediate treatment is offered to correct the position of their feet. In the Middle Ages however, no such treatment was available, and people were forced to find other means to ambulate.

Besides the disability of his feet, his left foot shows seven toes, compatible with polydactyly. Since the paint of his right foot is damaged, the number of toes on that side can’t be examined. Furthermore, he is missing the distal phalanx of his right thumb. The combination of clubfeet and polydactyly is a very rare condition. It has also been reported in combination with a missing hallux. Some papers describe the associations of these disabilities with rare syndromes, such as homeobox PITX1 gene mutation and Werner mesomelic syndrome. There have been around 30 case reports on this topic [5]. Other possible diagnoses include trisomy 13 (Patau syndrome); a chromosomal disorder that often features polydactyly and can include club foot among other anomalies, and Laurence-Moon-Bardet-Biedl syndrome; a syndrome which features polydactyly and other skeletal abnormalities, sometimes involving the feet.

### To lavish the thirsty

On the right side of the second panel, a man and a woman are shown offering water. The man pours water with an unusually extended posture of his arm and shoulder angle. This could suggest a physical limitation, though it is more likely an artistic exaggeration, as he is a helper rather than one of the sick.

Most notably, the man receiving water on the left side of the panel draws particular attention. He is dressed in a pink garment and is depicted climbing a small set of stairs. His posture is markedly hunched, with a forward curvature centred around the thoracic spine; suggestive of a kyphotic deformity. He supports himself with a cane held in his left hand, indicating possible mobility limitations. Additional notable features include his bald head, disproportionately large body and head, and an abnormally shaped nose.

There are several possible medical explanations for his appearance. At the time of the artwork in the sixteenth century, infectious diseases were widespread due to the lack of effective preventive measures and availability of treatment. One relevant example is tuberculosis, a chronic infectious disease caused by *Mycobacterium tuberculosis* that can persist over a lifetime and affect various organs. Spinal tuberculosis, also known as Pott’s disease, is the most common cause of kyphotic spinal deformities worldwide [6]. This form of extrapulmonary tuberculosis often affects the vertebral column, particularly in the thoracic region, leading to osteonecrosis and ultimately collapse of one or two vertebral bodies, usually of the thoracic spine. The resulting deformity, known as Pott’s kyphosis, can cause not only significant cosmetic disfigurement but also respiratory complications, costo-pelvic impingement, and neurological deficits such as late-onset paraplegia [7].

Another potential diagnosis is syphilis, a sexually transmitted infection caused by *Treponema pallidum*, a spirochete bacterium. Advanced or congenital forms of syphilis can lead to a collapse of the nasal bridge, resulting in a so-called saddle nose deformity. This characteristic loss of cartilage often arises from chronic infection [8]. In rare cases, syphilis can also involve the vertebrae, contributing to spinal deformities similar to kyphosis. *Tabes dorsalis* is the result of syphilitic neurodegeneration and leads to sensory loss and walking impairment, explaining his need for a stick. Moth-eaten alopecia may be associated with secondary syphilis.

Additionally, the man’s pronounced facial features, such as the large head and broad facial structure, could point to acromegaly, a hormonal disorder most often caused by a pituitary adenoma. In cases of early-onset growth hormone excess, gigantism may occur, while in adults, acromegaly manifests as abnormal growth in the hands, feet, jaw, and soft tissues such as the tongue and nose [9]. His enlarged craniofacial proportions may support this hypothesis, although it remains speculative. Taken together, this figure appears to embody a convergence of visual signs that could reflect real pathological conditions observed in the early sixteenth century, whether as an intentional depiction of illness or a reflection of societal familiarity with such disabilities.

The person in the green garment, positioned in the foreground of the panel, is shown in a kneeling position. Attached to his lower legs are assistive slats, likely early forms of self-made mobility aids, suggesting that he is unable to walk without support. It appears that he lacks both feet after amputation. Additionally, he supports himself with crutches placed under his arms. The condition of his lower legs is unclear, partially due to the limited visibility of this area in the composition. A possible explanation for this condition is poliomyelitis, an infectious disease caused by an enterovirus. Most cases are asymptomatic, but some may present with mild flu-like signs or, less commonly, neurological symptoms like neck stiffness or paraesthesia. In several cases, polio may cause lasting paralysis or death, and years later, post-polio syndrome can lead to renewed muscle weakness [10].

Another interesting diagnosis to be considered is ergotism, a condition caused by consuming rye or grain contaminated with *Claviceps purpurea*, a fungus commonly producing ergot alkaloids. During the Middle Ages, its pathogenesis was not recognized despite epidemic proportions of the disease. In regions where rye was a staple food, numerous cases of epidemics were documented and described as “Saint Anthony’s fire” or “holy fire” [11]. Historically, ergotism manifested in two forms. The first form is the convulsive type, this type involves symptoms such as muscle twitching, widespread seizures, and abnormal sensations like numbness or tingling. The second, gangrenous form is marked by severe inflammation, swelling, and a burning pain most often in the legs and feet. In such cases, the affected limbs typically became numb, darkened in color, progressively shriveled, and ultimately dried out, taking on a mummified appearance due to severe, peripheral vasoconstriction induced by the infection [12]. End-stage gangrene of the upper or lower extremities necessitated surgical amputation if the necrotic parts would not have fallen off spontaneously (auto-amputation). The use of additional assistive devices may further indicate neurological complications, which are associated with advanced stages of the disease. While the depicted devices would be considered unusual by modern standards, they offer a valuable glimpse into the types of prosthetic or supportive equipment that were in use during the early sixteenth century.

To the right of the central pair, a bearded man with a walking stick is missing his lower right leg. A bandage is wrapped around the stump at knee level, suggesting a relatively recent injury. The amputation could have been the result of trauma or infection, which was a common cause of limb loss before the advent of modern antibiotics and surgical techniques. Arterial obstructive disease at that time was quite unusual.

### Clothing the naked

The third panel depicts the act of clothing the naked. No physical abnormalities or signs of illness could be identified in this scene.

### Burying the dead

The central panel depicts the burial of the dead by members of the Brotherhood. Figures dressed in black are shown mourning the deceased. It is suggested that this scene refers to the burial of individuals who died from the plague. A small group of men dressed in black could be identified as the *Cellebroeders*, later known as the Alexians, who specialized in caring for and burying plague victims.

### Sheltering strangers

This panel depicts the act of sheltering travellers, in this case pilgrims [13]. Interestingly, in this scene it is not one of the persons in need but rather the man providing shelter, the figure in the red coat assisting the man in the blue cloak, who appears to show signs of an orthopaedic condition. His right lower leg is hyperextended; *Genu recurvatum*, a deformity that could be attributed to joint hypermobility or to trauma resulting in a rupture of the anterior cruciate ligament, or joint with more severely, damage to the meniscus or knee capsule [14]. Clinically, the hyperextended leg looks very much like this condition, but it could also have been an artistic exaggeration of the artist.

### Caring for the sick

The sixth panel shows an idealized interior of a hospital or a hospice. In reality, hospitals in the late Middle Ages were probably much more crowded and not every patient had access to a private bed. The patients do not appear to show any obvious physical disabilities, making it impossible to determine what illnesses or conditions they may be suffering from. None of the figures on the panel show attributes of a physician. As doctors often visited such institutions only a few times a week, the Brotherhood likely provided daily care [15].

### Visiting prisoners

The last panel shows prisoners confined within a prison. Members of the Brotherhood are shown visiting the inmates, paying money to secure the release of those deemed worthy of liberation. The person in the red garment in the foreground appears to have hyperextended knees, similar to the figure depicted in the fifth panel. While this may have been a stylistic choice by the artist, it nonetheless presents a clinical resemblance to hyperextension. No other disabilities or signs of illness are discernible in this panel.

**Table 1:** Possible orthopaedic diagnoses in *The Seven Works of Mercy*

Panel	Figure / Description	Clinical features	Possible underlying pathology/ diagnosis(es)
**Feeding the Hungry**	Man in blue garment, seated in foreground	- Clubfoot (*congenital talipes equinovarus*) - Polydactyly (seven toes on left foot) - Missing distal phalanx of right thumb	PITX1 mutation, Werner mesomelic syndrome, Patau syndrome, Laurence-Moon-Bardet-Biedl syndrome
**To Lavish the Thirsty**	Man offering water	Extended posture right arm	
	Central man in pink, climbing stairs	- Thoracic kyphosis - Saddle nose - Alopecia - Tabes dorsalis - Walking disability - Pronounced facial features: large head and broad facial structure	Pott’s disease / spinal tuberculosis, syphilis, acromegaly (enlarged skull and facial features)
	Man in green, foreground, with wooden leg aids		- Poliomyelitis (paralysis, leg weakness) - St. post bilateral (auto)amputation of feet due to ergotism (dry gangrene, neurological damage)
	Bearded man walking with a stick	St. post leg amputation	Trauma, infection, e.g., gangrene or wound complication
**Clothing the Naked**	—	No visible physical abnormalities identified	
**Burying the Dead**	—	Implied reference to plague victims, but no visible disabilities	
**Sheltering Strangers**	Man in red garment assisting pilgrim	Genu recurvatum (knee hyperextension)	Possibly due to ligament injury, trauma, or hypermobility
**Caring for the Sick**	—	No visible orthopaedic deformities; figures possibly ill but without external signs	
**Visiting Prisoners**	Man in red, foreground	Genu recurvatum (knee hyperextension)	Possibly due to ligament injury, trauma, or hypermobility

## Discussion

The *Seven Works of Mercy* is a unique masterpiece, offering a valuable insight into medical conditions and the societal perceptions of illness and disability in the early sixteenth century. The Master of Alkmaar depicted various physical abnormalities with such precision, that we can now, after 520 years, identify them. This is the first paper that systematically identifies these conditions in an effort to gain a deeper understanding of common orthopaedic disabilities occurring in that period. It is crucial, however, to consider artistic stylization of the artist. Therefore, while recognizing physical traits that align with clinically known, common illnesses and disabilities, we should be aware of the ambiguity in our observations and interpretations. The challenge of iconodiagnosis lies in the fact that each artwork represents a subject at one specific point in time. Researchers should keep in mind that accurate iconodiagnosis primarily relies on the observer’s eye and mind. Its effectiveness, however, depends on the examiner’s understanding of the artistic methods used to depict pathological features. In this article the diagnoses were carefully tested against the 11- step iconodiagnostic method [2]. The fourth step includes comparison with other works of the artist and could not be performed, as no other works by the artist are known. Step nine includes evaluation by a panel of medical experts and was carried out by medical specialists during tours at the Rijksmuseum.. The level of certainty for a diagnosis depended on the respective case and has extensively been discussed in this article.

**Fig. 2:**
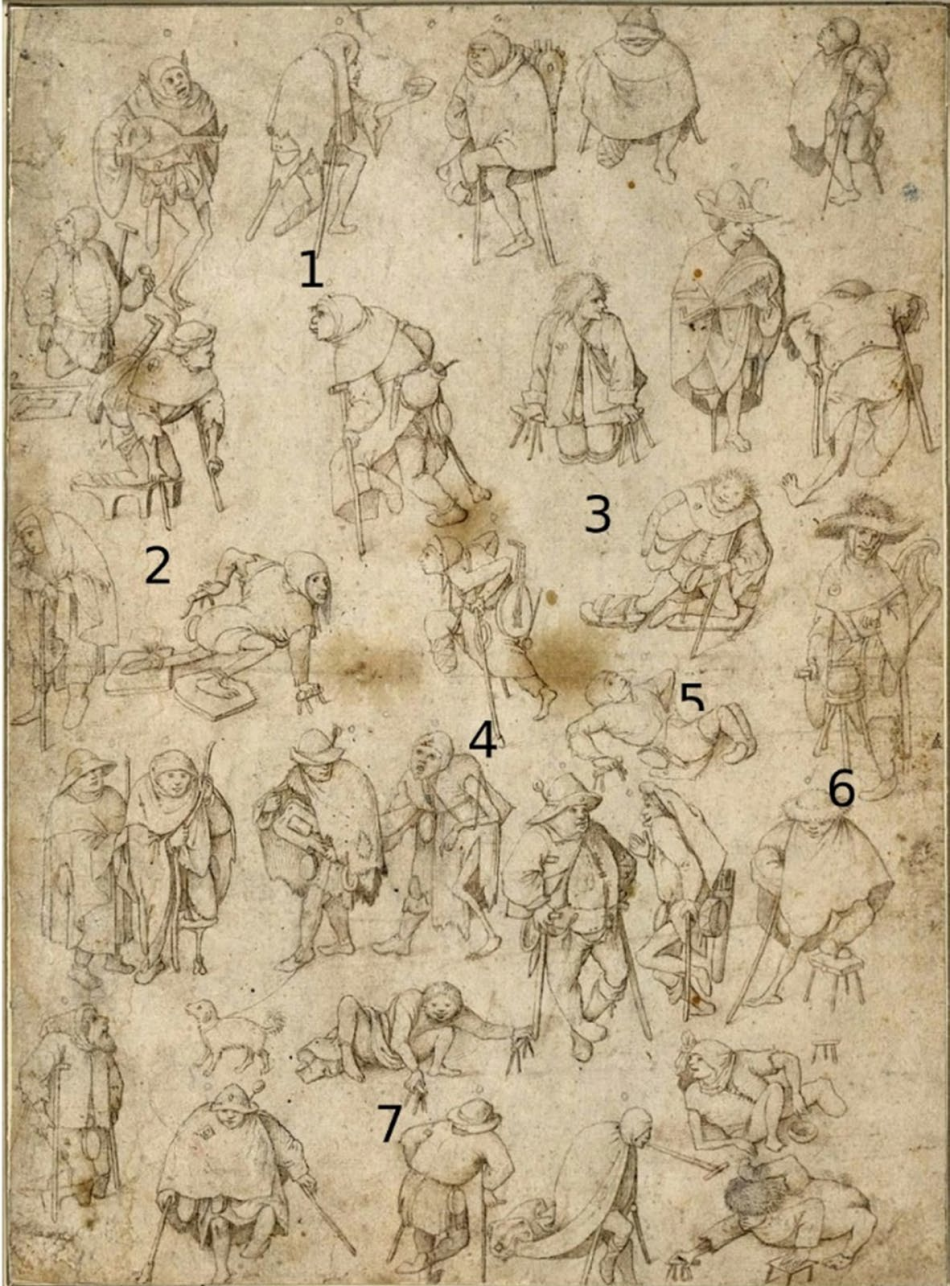
Hieronymus Bosch or a follower of his, *Procession of the Cripples*, ca. 1530-1540 (presumed), pen in brown ink, partially washed, on paper; circular markings in pencil, 28,5 × 20,8 cm, Albertina Museum, Vienna, public domain

**Fig. 3:**
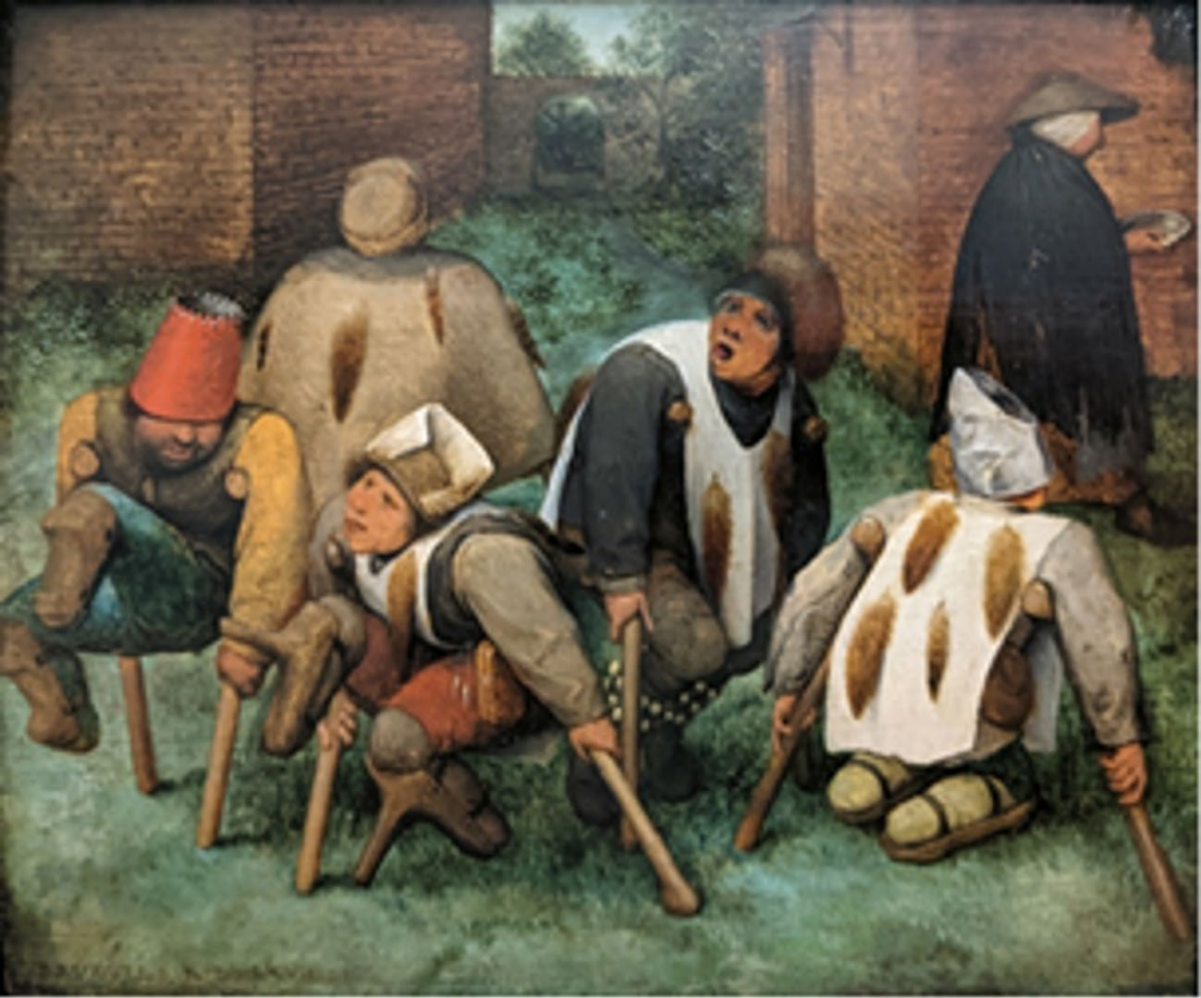
Pieter Bruegel the Elder, *The Beggars*, 1568, oil on panel, 18,5 × 21,5 cm, Louvre, Paris, public domain

The physical deformities of the cripples depicted in *The Seven Works of Mercy* can be compared to other disabled individuals portrayed in the same century with similar assistive devices and postures, for example in the works attributed to either Hieronymus Bosch or a follower of his (Figure 2) and Pieter Bruegel the Elder (Figure 3).

Figures 2 and 5 in Figure 2 carry orthopaedic devices (such as crutches and slats with bindings) for the lower legs, quite similar to the seated person in the foreground in the second panel of the Master of Alkmaar. Although the lower legs of the figure depicted in Figure 3 are not visible, the posture with hand crutches suggests a case of bilateral foot amputation because of ergot induced gangrene.

Similarly, in Bruegel’s painting, several amputees are depicted using various assistive devices, who probably had suffered from ergotism (Figure 3) [16].

## Conclusion

*The Seven Works of Mercy* by the Master of Alkmaar is not just a religious artwork, it mirrors society in a town in the early sixteenth century, in which several orthopaedic conditions are displayed. Obviously, the disabled members of the community were those in need of the benefits bestowed upon them by their fellow citizens, hence their presence in this artwork. While artistic interpretation must be considered and a definitive diagnosis cannot be made from art only, the depicted features suggest that close observation by the artist captured real orthopaedic pathologies which existed at the time of creation.

## Data Availability

No datasets were generated or analysed during the current study.
